# Structural snapshots of human DNA polymerase μ engaged on a DNA double-strand break

**DOI:** 10.1038/s41467-020-18506-5

**Published:** 2020-09-22

**Authors:** Andrea M. Kaminski, John M. Pryor, Dale A. Ramsden, Thomas A. Kunkel, Lars C. Pedersen, Katarzyna Bebenek

**Affiliations:** 1 Genome Integrity and Structural Biology Laboratory, National Institute of Environmental Health Sciences, National Institutes of Health, 111 TW Alexander Dr., Bldg. 101/Rm F338, Research Triangle Park, NC 27709 USA; 2 Curriculum in Genetics and Molecular Biology, University of North Carolina at Chapel Hill, 32-046 Lineberger Comprehensive Cancer Center, 450 West Dr., CB 7295, Chapel Hill, NC 27599 USA

**Keywords:** DNA, X-ray crystallography

## Abstract

Genomic integrity is threatened by cytotoxic DNA double-strand breaks (DSBs), which must be resolved efficiently to prevent sequence loss, chromosomal rearrangements/translocations, or cell death. Polymerase μ (Polμ) participates in DSB repair via the nonhomologous end-joining (NHEJ) pathway, by filling small sequence gaps in broken ends to create substrates ultimately ligatable by DNA Ligase IV. Here we present structures of human Polμ engaging a DSB substrate. Synapsis is mediated solely by Polμ, facilitated by single-nucleotide homology at the break site, wherein both ends of the discontinuous template strand are stabilized by a hydrogen bonding network. The active site in the quaternary Pol μ complex is poised for catalysis and nucleotide incoporation proceeds in crystallo. These structures demonstrate that Polμ may address complementary DSB substrates during NHEJ in a manner indistinguishable from single-strand breaks.

## Abstract (web summary)

Polymerase μ (Polμ) participates in the repair of DNA double-strand breaks (DSBs) via the nonhomologous end-joining (NHEJ) pathway. Here, the authors determine the crystal structure of a pre-catalytic ternary complex of human Polμ with a bound DSB substrate and they obtain further mechanistic insights by allowing the insertion reaction to proceed in crystallo, which enabled them to determine a Polμ structure with incomplete incorporation and the structure of the post-catalytic nicked state.

## Introduction

Genomic DNA is vulnerable to damage/breakage caused by exogenous exposures to ionizing radiation or endogenous reactive oxygen species generated through cellular metabolism^1^. When phosphodiester backbone breaks on opposing DNA strands cluster, cytotoxic DNA double-strand breaks (DSBs) form. Persistently unrepaired DSBs in DNA can have disastrous consequences, leading to human cancers and other diseases^2^. Alternatively, DSBs can be systematically generated in a programmed manner, as observed in the V(D)J recombination pathway required for immunoglobulin gene maturation^3^. DSBs are repaired by multiple pathways, including nonhomologous end-joining (NHEJ), which is favored in nonreplicating cells, or in cells lacking replicated sister chromatids^4^. The multiprotein NHEJ complex binds and bridges broken ends so they can be ultimately rejoined^5^. Its assembly is presumed to occur in a stepwise fashion, wherein the Ku70/80 heterodimer first identifies and binds the ends, in concert with the DNA-PK catalytic subunit. This core complex, known as the DNA-PK holoenzyme, enlists other protein binding factors—DNA Ligase IV, XRCC4, Artemis, XLF, and various polymerases—to subsequently process and rejoin the ends, depending on their sequence and structural composition^5^.

The Family X polymerases (Pols), Polλ, Polμ, and terminal deoxyribonucleotidyl transferase (TdT) are recruited to the NHEJ complex by means of an N-terminal BRCT protein–protein interaction domain^6^. All three Family X enzymes are involved in V(D)J recombination, though with distinct expression and polymerization profiles. Pols λ and μ are widely expressed, primarily template-dependent polymerases, which function in maturation of immunoglobulin heavy- and light-chain loci, respectively^7–9^. In contrast, TdT expression is strictly limited to immunological cells, where it functions as a primarily template-independent polymerase contributing to gene sequence diversity during V(D)J recombination^10^. In addition to their specific roles in V(D)J recombination, Pols λ and μ also function more broadly in classical NHEJ. While both polymerases can utilize complementary DSB substrates with paired primer termini, Polμ is uniquely capable of bridging broken DNA ends lacking break site microhomology^11,12^. Though there exists a plethora of biochemical^13–16^ and structural^17–21^ information illustrating the activity of these Family X polymerases on single-strand break (SSB) substrates, understanding how each individual polymerase copes with DSB end-bridging is unclear. Thus far, only DSB-bound crystal structures of murine TdT^22,23^ and a chimeric construct of murine TdT containing the Loop1 region from murine Polμ^24^ are currently available, providing an important yet incomplete portrait of Family X polymerase behavior during NHEJ. We therefore present high-resolution crystal structures of the human Polμ catalytic domain simultaneously engaging both ends of a complementary DSB substrate. A catalytically-poised pre-catalytic quaternary complex was trapped using a correctly paired nonhydrolyzable nucleotide, which could be exchanged with a hydrolyzable nucleotide to generate “snapshots” of the incorporation reaction proceeding in crystallo. These structures indicate that Polμ provides a rigid scaffold, which can accommodate both SSBs and complementary DSBs in a nearly indistinguishable manner.

## Results

### Pre-catalytic hPolμΔ2 quaternary complex with complementary DSB

**Fig. 1:**
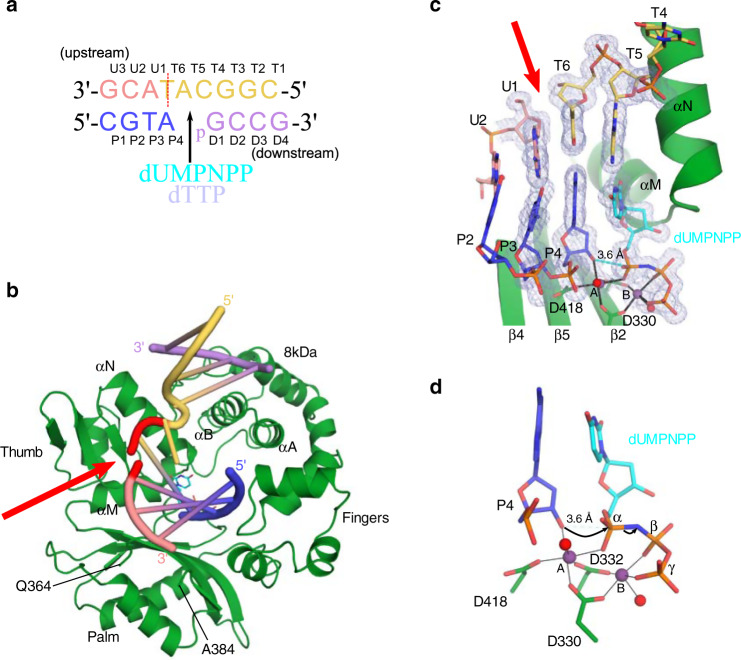
Structural characterization of hPolμΔ2 engaging a complementary DSB substrate. **a** DNA substrate crystallized with hPolμΔ2 and nonhydrolyzable dUMPNPP. Template strand discontinuity is marked (dashed red line). **b** Pre-catalytic quaternary DSB (protein, dark green; DNA and dUMPNPP colored as in **a**; template break site, red) The ordered ends of Loop1 are indicated (Gln364/Ala384). **c** hPolμΔ2 pre-catalytic quaternary complex active site. 2*F*_*o*_*–F*_*c*_ electron density (gray mesh) contoured at 1σ. Ionic interactions (Mg^2+^ ion, purple; water molecules, red) are indicated by black bars. Interatomic distance between 3′-OH and α-phosphate (dashed cyan line) was measured in PyMOL (Schrödinger). **d** Zoomed-in view of the hPolμΔ2 active center, with arrows indicating putative electron movements during reaction progression.

A variant of the Pol μ catalytic domain with increased crystallizability and biochemical characteristics equivalent to those of the wildtype enzyme was used for this study (henceforth, referred to as hPolμΔ2^21^). In this variant, the flexible, non-conserved loop (Loop2) between β-strands 4 and 5 of the palm subdomain was deleted (ΔPro398–Pro410), and the ends of the β-strands were fused by a single glycine residue (Gly410). The hPolμΔ2 pre-catalytic quaternary complex was incrementally assembled by first incubating the protein with an annealed downstream DNA duplex containing a 5′-phosphorylated downstream primer (Fig. 1a) to dictate correct binding, as Pol μ prefers to orient the nascent base pair binding site using the 5′-end of the gap rather than the 3′-end^20^, and its activity is stimulated by the presence of a phosphate at that position^15^. Synapsis is then achieved by the polymerase after addition of annealed upstream DNA—facilitated by 3′-primer terminal single-nucleotide complementarity (A:T), consistent with reports that Polμ efficiently mediates end-bridging without other NHEJ-binding partners^25^. Binding a correctly paired nonhydrolyzable nucleotide analog 2′-deoxyuridine-5′-[(α,β)-imido]triphosphate (dUMPNPP) in the active site leaves the enzyme poised for catalysis (PDB ID code 6WIC, Fig. 1b and Table 1). The upstream and downstream duplex regions are positioned distally from one another, an arrangement induced by an ~90° bend in the template backbone immediately downstream of the nascent base pair binding site. The upstream primer terminal sugar (residue P4) and that of the incoming dUMPNPP are observed with C3′-*endo* sugar puckers, which leaves the 3′-OH ideally positioned for in-line attack on the α-phosphate (3.6 Å) of dUMPNPP (Fig. 1c, d). Both divalent metal sites are occupied by Mg^2+^ ions, which are observed with octahedral coordination mediated by the 3′-OH, Asp330, Asp332, Asp418, the triphosphate oxygens, and associated water molecules.

**Table 1:** Data collection and refinement statistics.

	Pre-catalytic complex^a,b^	Incomplete incorporation^a,b^	Post-catalytic complex^a,b^
PDB ID code	6WIC	6WID	6WIE
*Data collection*			
Space group	P2_1_2_1_2_1_	P2_1_2_1_2_1_	P2_1_2_1_2_1_
*Cell dimensions*			
*a*, *b*, *c* (Å)	60.13, 62.23, 118.05	60.19, 62.04, 118.32	60.07, 62.25, 118.32
Α, β, γ (°)	90, 90, 90	90, 90, 90	90, 90, 90
*Resolution (Å)*	50–1.55 (1.58–1.55)^c^	50–1.50 (1.53–1.50)	50–1.50 (1.53–1.50)
*R*_sym_ (%)	6.2 (53.5)	8.3 (54.8)	10.6 (57.5)
*I*/σ*I*	26.63 (1.5)	28.47 (2.24)	18.54 (1.77)
Completeness (%)	99.5 (98.1)	99.8 (99.9)	99.8 (99.8)
Redundancy	6.4 (4.5)	6.0 (5.5)	6.3 (5.4)
*Refinement*			
Resolution (Å)	35–1.55	31.0–1.50	35.0–1.50
No. reflections	64,729	71,176	71,906
*R*_work_/*R*_free_ (%)	16.55/17.94	15.70/16.95	16.99/18.68
Incorporation extent	0%	40%	100%
*No. of atoms*			
Protein	2563	2562	2564
DNA	379	420^d^	399
Nucleotide	28^e^	28^e^/9^f^	9^g^
Water	347	348	346
*B-factors*			
Protein	22.89	18.38	20.17
DNA	19.06	13.87^d^	15.45
Nucleotide	14.20^e^	9.21^e^/14.56^f^	25.08^g^
Water	33.38	31.09	31.04
*R.m.s. deviations*			
Bond lengths (Å)	0.009	0.009	0.009
Bond angles (°)	1.038	1.087	1.030

^a^A single crystal was used to collect each data set.

^b^These crystals were collected on the Southeast Regional Collaborative Access Team (SER-CAT) 22-ID beamline at the Advanced Photon Source at Argonne National Laboratory.

^c^Values in parentheses are for highest-resolution shell.

^d^Includes atoms from unincorporated and incorporated alternate conformations of residues P4 and P5.

^e^Nonhydrolyzable incoming dUMPNPP nucleotide.

^f^Inorganic pyrophosphate leaving group.

^g^Partially disordered inorganic pyrophosphate leaving group.

### Protein–DNA interactions in the hPolμΔ2 quaternary complex

**Fig. 2:**
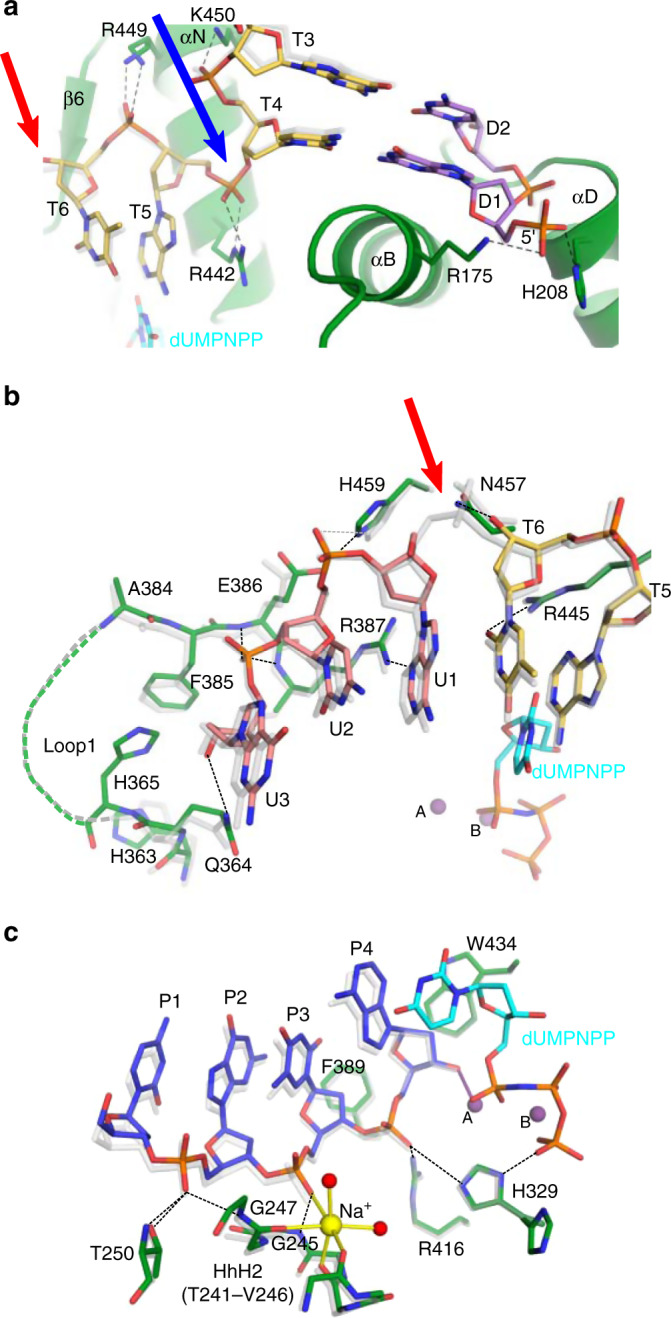
Comparison of hPolμΔ2 interactions with SSB or complementary DSB substrates. Superposition of hPolμΔ2 pre-catalytic complexes bound to a DSB (colored as in **1a**) or a 1nt-gapped SSB (gray, PDB ID code 4M04^21^). Interactions involving downstream duplex **a**, template strand **b**, and upstream primer strand **c** near the break site are diagrammed (Table 2). Putative hydrogen bonds in DSB and SSB complexes drawn as dashed lines (black or gray, respectively). Ionic interactions (solid lines) are color-coordinated with metal identity. Red and blue arrows indicate template backbone break and bend, respectively. Arg449 and Arg175 sidechains in **a** are partially disordered and only the ordered regions are included in the model.

From hPolμΔ2’s perspective, the process of binding the downstream duplex to the 8 kDa subdomain likely occurs in a similar fashion, regardless of whether the DNA substrate contains an intact or discontinuous template strand, as relevant breaks occur upstream of the nascent base pair binding site. The ~90° bend in the template backbone between residues T4 and T5 opens the helix to allow access of incoming nucleotides to the nascent base pair binding site, positioning the 5′-phosphate on the downstream primer >20 Å from that location (Fig. 2a). This bend is reinforced by multiple putative hydrogen bonding interactions from the nonbridging phosphate oxygens of residues T4–T6 to the sidechains of Arg442, Arg449 (partially disordered), and Lys450 (Table 2). The position of the downstream duplex is stabilized by van der Waals interactions with the backbone of Gly174 on the N-terminal end of α-helix B, anchoring the 5′-phosphate to the 8 kDa subdomain via putative hydrogen bonds with the sidechains of Arg175 (partially disordered) and His208 (Fig. 2a and Table 2). Interestingly, mutations of Gly174 and Arg175, which diminish the fidelity and efficiency of Polμ’s activity in NHEJ^26^, have been discovered in skin and ovarian cancers, respectively^27,28^.

**Table 2:** Putative hydrogen bonding interactions in the hPolμΔ2 pre-catalytic quaternary complementary DSB complex.

Region	Interacting atoms	Interatomic distance^a^ (Å)
Downstream primer strand	Arg175^b^ NE–D1 OP1	3.3
	His208 ND1–D1 OP2	2.6
	Ser209 N–D2 OP1	3.0
	Gly206 N–D2 OP1	3.0
	His208 N–D2 OP2	3.0
	Glu207 N–D2 OP2	3.3
	His204 N–D3 OP1	2.9
Downstream template strand	Arg449^b^ NE -- T6 OP1/2	2.6–3.3
	Arg442 NE–T5 OP2	2.9
	Arg442 NH2–T5 OP1	3.0
	Ly450 NZ–T4 OP1	2.6
	Asn457 ND2–T6 O3′	2.9
	Arg445 NH1–T6 O2	2.9
Upstream template strand	Arg387 NH1–U1 N3	3.0
	Glu386 N–U3 OP1	2.8
	Arg387 N–U3 OP1	3.0
	Glu364 NE2–U3 O3′	3.1
Upstream primer strand	Thr250 N–P2 OP1	3.1
	Thr250 OG1–P2 OP1	2.7
	Gly247 N–P2 OP1	2.9
	Gly245 N–P3 OP1	2.9
	Arg416 NH2–P4 OP1	2.8
	His329 NE2 (A)–P4 OP1	3.6

^a^Putative hydrogen bonds were assessed based on geometry and interatomic distances measured in PyMOL.

^b^Measured distances are approximate, since sidechains are partially disordered.

Electron density for the upstream template strand clearly highlights the backbone discontinuity between residues T6 and U1 (Fig. 1c). Both sides of the break in the pre-catalytic complex are stabilized by a hydrogen bonding network within Polμ’s substrate binding cleft (Fig. 2b and Table 2). The O5′ atom on upstream template residue U1 is solvent-exposed, but the O3′ atom on the 3′-end of downstream template residue T6 orients toward the protein surface and putatively hydrogen bonds with Asn457. Arg445 also lies in the minor groove within hydrogen bonding distance of the T6 base. Arg387 interacts with the U1 base upstream of the break. The U2–U3 phosphate lies within hydrogen bonding distance of the Glu386 and Arg387 backbone amide nitrogens, whereas Gln364 putatively interacts with the 3′-OH of residue U3.

Upstream primer strand positioning is stabilized by van der Waals, ionic, and hydrogen bonding interactions (Fig. 2c and Table 2). The aromatic sidechains of Trp434 and Phe389 likely stabilize the sugar moieties of the primer terminal and penultimate nucleotides, respectively, via π-CH interactions. The upstream P1–P2 phosphate is secured by multiple interactions between its nonbridging oxygens and the Thr250 sidechain, its backbone amide, and the backbone amide of Gly247. The P2–P3 phosphate is stabilized by a putative hydrogen bond with the backbone amide of Gly245 and an interaction with the Na^+^ coordinated by the HhH2 (helix-hairpin-helix) motif (residues Thr241–Val246) that is conserved throughout the Family X polymerases^6^. The P3–P4 phosphate is tethered near the catalytic center via a hydrogen bond with Arg416, which has been shown to be dispensable for nucleotide incorporation on SSBs but essential for DSB repair^29^. The His329 sidechain, thought to bridge the primer terminal phosphate and incoming nucleotide during NHEJ^19^, is observed in multiple conformations, one of which lies within long-range hydrogen bonding distance of a P3–P4 phosphate oxygen. Correct positioning of the primer terminal 3′-OH is crucial for catalysis and is largely mediated by coordination with the catalytic Mg^2+^.

### The observed hPolμΔ2 conformation is catalytically competent

**Fig. 3:**
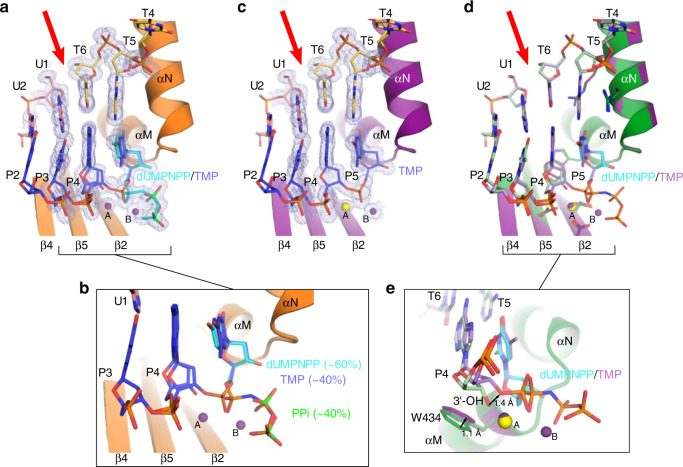
Catalytically competent hPolμΔ2 active site geometry leads to nucleotide incorporation in crystallo. **a** Structural snapshot of the hPolμΔ2 active site, exhibiting incomplete incorporation of the hydrolyzable dTTP nucleotide, with a zoomed view **b** of the primer terminal and unincorporated dUMPNPP (cyan) or newly incorporated TMP nucleotides. Protein components are shown as an orange ribbon; DNA is drawn in stick (colored as in Fig. 1a–b). Mg^2+^ ions are shown as purple spheres. The pyrophosphate (PPi) leaving group is drawn in green. **c** Fully post-catalytic nicked complex. Though Mg^2+^ fully occupies the B site in the post-catalytic complex, the A site metal has been partially replaced by Na^+^ (yellow). 2*F*_*o*_*-F*_*c*_ electron density for active site components is shown as a gray mesh (contoured at 1σ). Red arrow indicates template backbone break. Superposition of hPolμΔ2 pre- (green; dUMPNPP in cyan) and post-catalytic nicked (purple; Na^+^ ion in yellow) complexes, shown as global **d** or zoomed in **e** views. Positional shifts are shown by black arrows, with the measured distances indicated.

Transferring the hPolμΔ2 pre-catalytic quaternary complex crystals from a cryoprotectant solution containing nonhydrolyzable dUMPNPP to a cryoprotectant solution containing hydrolyzable dTTP allows nucleotide exchange and insertion in crystallo, yielding a structure exhibiting incomplete (~40%, PDB ID code 6WID, Table 1 and Fig. 3a–b) or nearly complete (PDB ID code 6WIE, Table 1 and Fig. 3c) incorporation after a longer soak. Successful nucleotide incorporation within the crystalline lattice indicates that the observed pre-catalytic quaternary conformation is indeed catalytically competent. Superposition of pre- and fully post-catalytic complexes reveals no large-scale movements of protein subdomains, DNA substrate, or active site residues during nucleotide insertion (0.084 Å RMSD over 278 Cα atoms, Fig. 3d). There is a slight (1.4 Å) shift of the 3′-primer terminus toward the α-phosphate of the newly incorporated nucleotide, allowing phosphodiester bond formation. A correlated adjustment (≤1.1 Å) of the Trp434 sidechain is also observed, mediated by a rotation around the CB-CG bond (Fig. 3e). These subtle motions appear to be a normal consequence of nucleotide insertion by Polμ^21^ and are likely observed regardless of DNA substrate configuration. There is no observed density for a third “product metal” in these structures (Fig. 3), even in the structure exhibiting incomplete incorporation where the “product metal” might be expected, which is consistent with previously published reports^30^. Upon reaction completion, the pyrophosphate leaving group becomes partially disordered and replaced by solvent molecules, which cannot be definitively modeled.

### 5′-phosphate binding becomes critical on tenuous DNA substrates

**Fig. 4:**
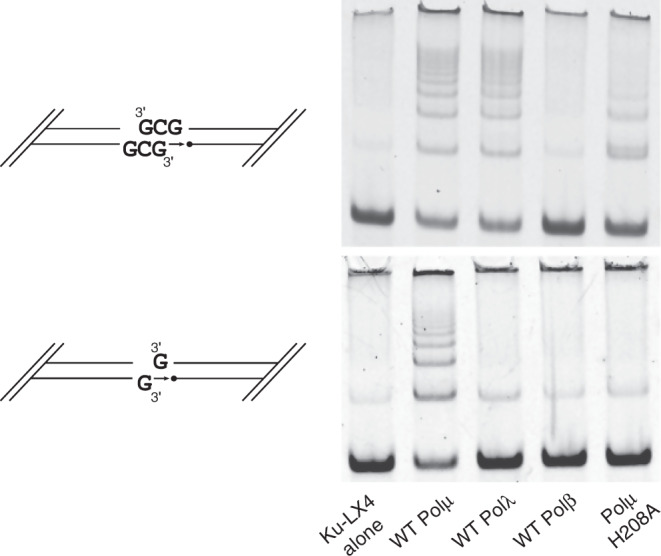
Qualitative assessment of the role of His208 in NHEJ proficiency. Activity of full-length wildtype hPolμ versus the Polμ H208A mutant in in vitro NHEJ concatamerization assay using complementary (top) or noncomplementary (bottom) DSB ends, as depicted in cartoons beside each panel. Downstream primer is 5′-phosphorylated (black circle). Pols λ and β are included for reference, as Pol β shows no detectable activity for either substrate, whereas Pol λ is active only on the partly complementary DSB. Source data are provided as a Source Data file.

Previous studies have shown that interactions with or near the 5′-phosphate on the downstream duplex can have deleterious consequences for efficiency and fidelity of end-joining activity by Polμ^26,31^. Disease-associated mutations of Gly174 and Arg175 demonstrated only subtle differences in polymerization efficiency on a single-nucleotide SSB substrate, which became more pronounced as the SSB became a complementary DSB. Repair of a noncomplementary DSB, which lacks microhomology at the break site to facilitate synapsis, was abrogated further^26^. Moreover, substitution of Arg175 with histidine showed less-deleterious effects on end-joining than did replacement with alanine^31^. In order to determine whether His208 (Fig. 2a) binding to the 5′-phosphate is similarly required for contribution of Polμ to end-joining, we generated an alanine substitution at this position and assayed its ability to mediate single-nucleotide gap-filling on both partly complementary and noncomplementary DSB ends. Similar to previous reports of the Gly174 and Arg175 mutants, the H208A mutant demonstrated decreased end-joining efficiency on complementary DSB ends (Fig. 4, top), compared with the wildtype enzyme. Activity of this mutant was negligible when compared with a no-polymerase control when using noncomplementary overhangs (Fig. 4, bottom).

## Discussion

**Fig. 5:**
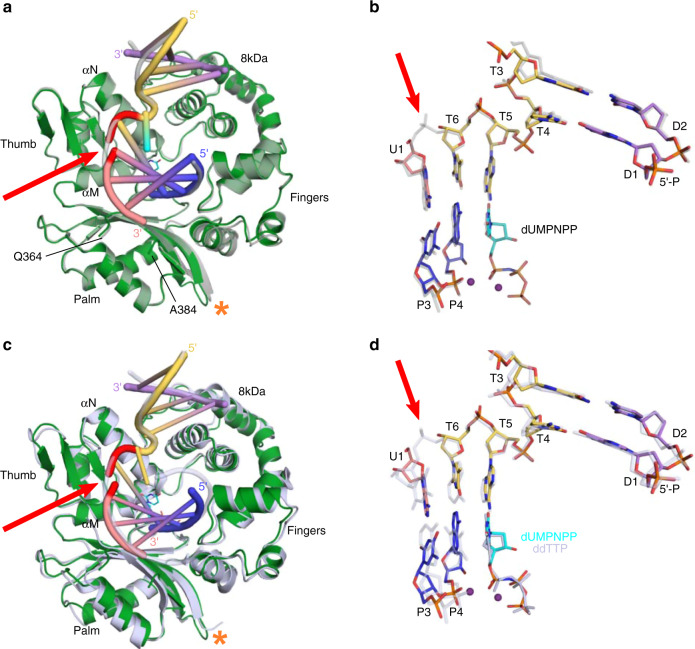
Comparison of the hPolμΔ2 complementary DSB and other human or mouse Polμ crystal structures. Superposition of hPolμΔ2 complementary DSB (colored as in Fig. 1a–b) with **a** hPolμΔ2 (gray, PDB ID code 4M04^21^) or **c** mouse Polμ (light blue, PDB ID code 2IHM^19^) 1nt-gapped SSB substrates, with zoomed-in view of superimposed human **b** or mouse **d** DNA substrates in stick. Red arrow and orange asterisk indicate the location of the broken template backbone and the hPolμΔ2 Loop2 deletion, respectively.

Superposition of the human hPolμΔ2 pre-catalytic quaternary complementary DSB and either the human (Fig. 5a, b and Fig. 2) or mouse (Fig. 5c, d) Polμ ternary SSB complexes reveal a high degree of global similarity (0.34 Å RMSD over 284 Cα atoms and 0.81 Å over 287 Cα atoms, respectively). Subtle differences can be observed in the position of the Loop2 region (disordered in mouse Polμ and deleted ΔPro389-Pro410 in the human Polμ) and at the distal ends of the DNA duplex, all of which can be influenced by crystal packing and DNA pseudo-stacking between the crystal forms. Interactions between protein and either the 5′-phosphate (Fig. 2a) or upstream primer strand (Fig. 2c) are conserved between both substrate types in these structures, but slight variations are observed in residues surrounding the break in the human structures, namely the sidechain conformations of Asn457 and His459 (Fig. 2b). His459 lies within long-range hydrogen bonding distance of the T7-T8 phosphate oxygens in the SSB (3.3 Å), but likely interacts instead with Glu386 (2.7 Å) on the end of β-strand 4 near Loop1 in the DSB complex. The precise nature of these interactions and their contributions to catalysis by Polμ in SSB and DSB repair are currently unclear, as previous studies have shown that glycine substitution of His459 had no apparent effect on single-nucleotide gap-filling on either SSB or DSB substrates, and the N457D mutant was profoundly impaired on all substrates^32^. The similarities between the single- and DSB complexes therefore suggest that Polμ addresses DSB substrates containing break site complementarity in a manner analogous to its engagement of gapped SSB substrates, and that the polymerization reaction proceeds through the same mechanism.

**Fig. 6:**
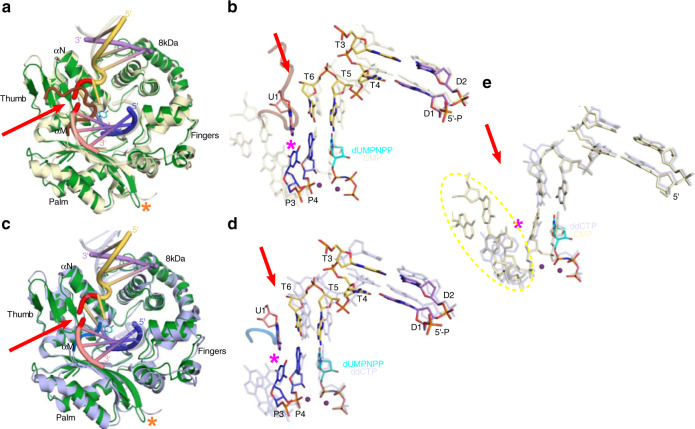
Comparison of the hPolμΔ2 complementary DSB and mouse TdT crystal structures. Superposition of hPolμΔ2 complementary DSB (colored as in Fig. 1a–b) with mouse TdT DSB synapse with **a** nicked complementary DSB substrate (khaki, PDB ID code 5D46^23^) or a **c** single-nucleotide complementary DSB substrate otherwise lacking an upstream template (light blue, PDB ID code 4QZ8^22^), with zoomed-in view of superimposed DNA substrates **b**, **d**. Loop1 residues from TdT are drawn in cartoon (brown and blue, respectively). **e** Superimposed DNA substrates from the TdT crystal structures, colored as in **c**, **d** to highlight the “wedged” upstream residues (dashed yellow circle). Red arrow indicates the location of the broken template strand. The magenta and orange asterisks indicate the positions of the “wedged” upstream residues and the hPolμΔ2 Loop2 deletion, respectively.

Comparison of the hPolμΔ2 quaternary complementary DSB structure with those available for mouse TdT reveals distinct differences. Superposition of the hPolμΔ2 complementary DSB with that of mouse TdT bound to a DSB substrate of similar configuration (PDB ID code 5D46^23^) shows structural correlation for the protein (RMSD of 1.04 Å over 275 Cα atoms, Fig. 6a), but less for the DNA (Fig. 6b). Equivalent residues comprising the primer terminus/nascent base pair binding site (referred to as an A-form “mini-helix” in an otherwise B-form duplex), and the downstream duplex show very similar positioning, but duplex upstream of the primer terminal base pair (P4:T6 in hPolμΔ2) is “wedged” open in mouse TdT by insertion of Loop1 residues into the duplex. A similar phenomenon is observed in another mouse TdT DSB synaptic complex mediated by single-nucleotide complementarity on the template strand (PDB ID code 4QZ8^22^, RMSD of 1.1 Å over 280 Cα atoms, Fig. 6c, d). The upstream primer strand in this structure is also “wedged” open, in the absence of an upstream template strand, suggesting that this behavior occurs independently of upstream base pairing in TdT (Fig. 6e). The “wedging” phenomenon is not observed in the hPolμΔ2 complementary DSB substrate.

**Fig. 7:**
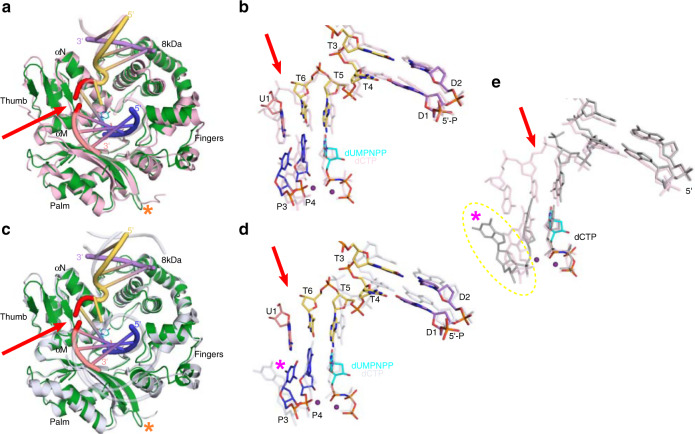
Comparison of the hPolμΔ2 complementary DSB and structures of the mouse TdT chimera harboring the Polμ Loop1 region. Superposition of hPolμΔ2 complementary DSB (colored as in Fig. 1a–b) with mouse TdT **a** 1nt-gapped SSB (light pink, PDB ID code 6GO5^24^) or **c** synapse with noncomplementary DSB (light gray, PDB ID code 6GO7^24^) substrates, with zoomed-in view of superimposed DNA substrates **b**, **d**. **e** Superimposed DNA substrates from the chimeric TdT-μ crystal structures, colored as in **c**, **d**, to highlight the “wedged” upstream residues (dashed yellow circle). Red arrow indicates the location of the broken template strand. The magenta and orange asterisks indicate the positions of the “wedged” upstream residues and the hPolμΔ2 Loop2 deletion, respectively.

A recent study of a chimeric form of mouse TdT harboring the Loop1 region from mouse Polμ (henceforth, referred to as TdT-μ) included crystal structures of the chimera in complex with a 1nt-gapped SSB substrate (PDB ID code 6GO5^24^, Fig. 7a, b) and with a noncomplementary DSB synaptic complex reinforced by a triplex-forming oligonucleotide (PDB ID code 6GO7^24^, Fig. 7c, d). Superposition of the hPolμΔ2 complementary DSB synaptic complex with these structures yielded some intriguing differences. As with the superpositions with TdT, comparisons of the TdT-μ chimeric complexes showed similarities in global protein structure (1.02–1.03 Å over 274–276 Cα atoms), but some differences in DNA structure. The TdT-μ crystal structure with the 1nt-gapped SSB shows strong similarity of the DNA substrate position with that of the hPolμΔ2 complementary DSB, and the “wedged open” upstream duplex is not observed in either structure (Fig. 7b). In the TdT-μ noncomplementary DSB, however, the 3′-terminal base of the downstream template strand—which could mispair with the upstream primer terminal base, but is instead disordered—leaving an unpaired primer terminus (Fig. 7d, e). As in the crystal structures of TdT, the duplex upstream of the primer terminal nucleotide is observed in a “wedged” open conformation, though not by direct intervention of the chimeric mouse Polμ Loop1 residues, as occurs in wildtype TdT. It is therefore tempting to speculate that Polμ might utilize different modes of substrate binding, based on the sequence and structure configuration at the break site. Opening of the upstream duplex in a noncomplementary DSB could provide more access of the Loop1 region to interact with and stabilize the broken template strand, but may not be required for repair of complementary DSBs.

**Fig. 8:**
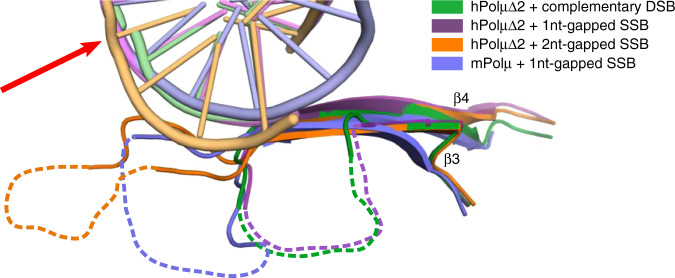
Variability in Polμ Loop1 conformations. Superpositions of mouse Polμ SSB (protein in blue, DNA in light blue, PDB ID code 2IHM^19^), and human PolμΔ2 SSB (1nt-gapped SSB with protein in purple, DNA in lavender, PDB ID code 4M04^21^; 2nt-gapped SSB in orange, DNA in light orange, PDB ID code 4YD1^20^) with the hPolμΔ2 complementary DSB structure (protein in green, DNA in light green, PDB ID code 6WIC). β-strands 3 and 4 are drawn, with the ordered ends of Loop1 illustrating its projected trajectory. The disordered regions of Loop1 are hypothetically modeled by dashed lines in colors correlated with the protein structure. Red arrow indicates the location of the broken template strand in the complementary DSB.

Among the Family X polymerases, the Loop1 motif is not required for repair of complementary DSB ends, but is critical for repair of DSBs lacking microhomology^12^. That Loop1 is disordered in these structures is perhaps unsurprising, given that repair of DSB substrates containing as little as one complementary base pair at the break site is Loop1-independent. Loop1 is largely disordered in all available structures of human or mouse Polμ—which contrasts with structures of TdT engaging a similar complementary DSB substrate, wherein an ordered Loop1 contributes to break site interactions (Fig. 6b)^23^. Comparison of the Loop1 conformations in the Polμ structures, however, may provide some insight. Superposition of the hPolμΔ2 1nt-gapped SSB and complementary DSB complexes shows that, although Loop1 is disordered in both structures, its overall trajectory and extent of disorder are similar (Fig. 2a and Fig. 8, green and purple dashed lines). In contrast, however, the overall trajectory of Loop1 in the human versus the mouse Polμ SSB structures widely differ (Fig. 8, purple and blue dashed lines, respectively). Loop1 also displays different conformations in the human Polμ complexes with different SSB substrates (1nt-gapped SSB, purple and 2nt-gapped SSB, orange). Although we cannot rule out the possibility that its conformations are influenced by crystal packing interactions, Loop1 likely adopts a conformation capable of stabilizing the template strand of any given substrate configuration, which becomes essential as the extent of break site complementarity decreases.

Since the Family X polymerases must contend with both ends of their substrates, often separated by >20 Å distance owing to the ~90° bend (Fig. 2a) in the template backbone, strong interactions with the 5′-phosphate, the primer terminus, and template strands are required for correct catalytically-competent geometry. This theory is supported by a variety of mutagenesis experiments, wherein different substrate interactions have been removed and interrogated for their roles in subsequent repair. Alanine substitution mutagenesis of Arg416^29^ and His329^19^ are thought to hinder correct positioning of the primer strand for catalysis (Fig. 2c). Likewise, loss of 5′-phosphate identification and positioning owing to mutations of Gly174^26^, Arg175^31^, or His208 (Fig. 4) lead to diminished repair capacity. These mutations have increasingly profound effects as the complexity of the repair substrate increases—SSB < complementary DSB < noncomplementary DSB. These results suggest that substrate binding by Polμ is multifactorial, with myriad interactions working in synergy. In more simplistic DNA substrate configurations, interactions in each key area (primer strand, 5′-phosphate, etc) serve a redundant role for the others. However, as synapsis becomes more tenuous, each individual interaction becomes critical. The DSB-bound Polμ structures presented in this study provide a greater understanding of how Polμ addresses different substrate configurations, and how interactions with these substrates contribute to DSB repair in NHEJ.

## Methods

### Expression and purification of human Polμ constructs

Full-length human Polμ and a crystallization variant of the human Polμ catalytic domain (hPolμΔ2; Pro132-Ala494 with Pro398–Pro410 of Loop2 deleted and replaced by Gly410) were cloned into the pGEXM vector^21^ and expressed in Rosetta2 (DE3) cells. hPolμΔ2 was expressed in Rosetta2 (DE3) cells in LB medium supplemented with 100 μg mL^−1^ ampicillin and 35 μg mL^−1^ chloramphenicol. Cells were grown at 37 °C to an OD_600nm_ of 0.8, at which point the temperature was decreased to 18 °C for ~30 minutes. Protein expression was induced by addition of isopropyl-β-d-thiogalactoside (IPTG) to a final concentration of 0.4 mm, and continued overnight at 18 °C. The cells were pelleted and resuspended in lysis buffer (25 mm Tris pH 8, 500 mm NaCl, 5% glycerol, 1 mm DTT) supplemented with 1 mm phenylmethylsulfonyl fluoride (PMSF) and cOmplete EDTA-free protease inhibitor tablets (1 tablet per 40 mL volume of lysis buffer). The cells were lysed by sonication and the lysate was subsequently cleared by centrifugation. Soluble protein was bound in-batch to glutathione 4B–Sepharose resin and subjected to on-resin TEV cleavage overnight at 4 °C. Polμ was then purified by size-exclusion chromatography on a Superdex200 26/600 column, equilibrated with lysis buffer. hPolμΔ2 was then further purified by ion-exchange chromatography, using a Mono Q 5/50 GL column equilibrated in the final storage buffer specific for each construct. The purified protein did not bind the column and was observed in the flow-through. Purified full-length Polμ was concentrated in 25 mm Tris pH 8, 100 mm NaCl, 5% glycerol, 1 mm DTT, and 10 mm MgCl_2_ buffer. The crystallization variant was concentrated in 25 mm Tris, pH 8, 75 mm NaCl, 5% glycerol, and 1 mm DTT. Purified proteins were flash frozen in liquid nitrogen and stored at −80 °C.

### hPolμΔ2 co-crystallization with DSB substrate and incoming dUMPNPP

The following DNA oligonucleotides (Integrated DNA Technologies) were used to generate the complementary DNA DSB substrate: upstream template (5′-ACG-3′), upstream primer (5′-CGTA-3′), downstream template (5′-CGGCAT-3′), and a 5′-phosphorylated downstream primer (5′-pGCCG-3′). Oligonucleotides comprising the upstream and downstream duplexes were separately mixed in equimolar ratios in 100 mm Tris, pH 7.5, and 40 mm MgCl_2_. Upstream and downstream DNA mixtures were separately annealed in a thermal cycler by denaturation at 94 °C, followed by a slow temperature gradient from 90 °C to 4 °C. The annealed DNA was then serially mixed in a 3:1 molar ratio with concentrated hPolμΔ2 (7.5–11.3 mg mL^−1^)—first the downstream DNA, followed by the addition of the upstream DNA, and finally the incoming dUMPNPP nucleotide (0.91 mm final concentration). The complex was incubated on ice at 4 °C for 1 h after each addition. Crystals of the quaternary complex were grown at 4 °C, by mixing equal volumes of complex and mother liquor (40–45.5 mm MES pH 5.6, 0.16–0.182 m KCl, 8.2–9.1 mm MgSO_4_, 8.2–9.1% w/v PEG400) using the sitting-drop vapor diffusion technique^33^. Crystals were transferred to a cryoprotectant solution containing 40 mm MES pH 5.5, 50 mm NaCl, 12 mm MgCl_2_, 0.1 m KCl, 30% w/v PEG400, 8 mm MgSO_4_, 10% glycerol, 1 mm dUMPNPP in two steps (PDB ID code 6WIC). The crystals were then flash frozen in liquid nitrogen and placed into a stream of nitrogen gas cooled to −180 °C for data collection.

### Nucleotide exchange and incorporation in crystallo

For the post-catalytic complexes, quaternary complex crystals were transferred from cryoprotectant solution containing 1 mm dUMPNPP to cryoprotectant solution containing 10 mm dTTP and soaked for 22 hours (PDB ID code 6WID) or 47 hours (PDB ID code 6WIE) at 4 °C. The crystals were then flash frozen in liquid nitrogen and placed into a stream of nitrogen gas cooled to −180 °C for data collection.

### Structure solution and refinement

Data were collected on the Southeast Regional Collaborative Access Team (SER-CAT) 22-ID beamline at the Advanced Photon Source (APS) at Argonne National Laboratory. The data were integrated and scaled using HKL2000^34^. The crystal structure of the 1nt-gapped SSB ternary complex with an incoming nonhydrolyzable analog (incoming dUMPNPP opposite template dA, PDB ID code 4M04^21^) was used as the search model for molecular replacement in Phaser^35^. The same *Rfree* test reflections were used for all structures to avoid potential model bias. All structures were refined by iterative cycles of manual model building and refinement in COOT^36,37^ and Phenix^38^. TLS (Translation/Libration/Screw) vibrational motion refinement^39^ was used for all structures. Data collection and refinement statistics are listed in Table 1. Ramachandran statistics were generated using MolProbity^40^.

### NHEJ assays

pFASTBAC1 constructs of human Ku70H6 (C-terminal hexahistidine tag), Ku83, hLigIVH6, and XRCC4^41^ were used to prepare baculovirus isolates for expression. All of the above constructs are available through Addgene. Hi5 cells were co-infected with isolates of either hLigIVH6 and XRCC4 (1:1 ratio) or Ku heterodimer (1:2 ratio of Ku70H6 to Ku83) constructs. Protein extracts were obtained in 50 mm sodium phosphate pH 8.0, 1 m KCl, 10% glyercol, 0.25% Triton X-100, 10 mm imidazole, and 7 mm β-mercaptoethanol, loaded onto a Ni-NTA Superflow column, and step-eluted with extraction buffer containing 350 mm imidazole. The LigIV-XRCC4 and Ku complexes were dialyzed against 25 mm Tris pH 8, 150 mm KCl, 10% glycerol, and 2 mm DTT (Buffer A), bound to a Mono Q HR 5/5 column, and eluted using 20 column volumes (CV) linear gradient with 25 mm Tris pH 8, 400 mm KCl, 10% glycerol, 1 mm DTT (Buffer B). Purified Ku and LigIV-XRCC4 complexes were dialyzed against Buffer B for storage.

pRSET-B-hPolλ^42^ was expressed in BL21-CodonPlus(DE3)-RIL cells, grown in LB medium at 28 °C to an OD_600nm_ of 0.5. Protein expression was induced by addition of 1 mm IPTG, followed 20 minutes later by 120 μg mL^−1^ rifampicin, and continued for 2 hours at 28 °C. Cells were pelleted by centrifugation, weighed, and frozen at −20°C. The frozen cell pellets were thawed and ground with 5.5-fold wet-weight of alumina in Buffer C (25 mm Tris pH 7.5, 1 m NaCl, 10% glycerol, 0.5 mm EDTA, 1 mm DTT) for 20 minutes at 4 °C. The suspension was clarified by centrifugation. DNA contamination was removed by precipitation with 0.3% polyethyleneimine and a second centrifugation step. The resulting supernatant was diluted four-fold to 250 mm NaCl with Buffer D (25 mm Tris pH 7.5, 10% glycerol, 0.5 mm EDTA, 1 mm DTT) and precipitated with ammonium sulfate to 65% saturation. The pellet was resuspended with Buffer D supplemented with 50 mm NaCl and loaded onto a PC column equilibrated with the same buffer. After copious washing with Buffer D supplemented with 100 mm NaCl, bound Polλ protein was eluted with Buffer D supplemented with 200 mm NaCl. The eluate was diluted with an equal volume of Buffer E (50 mm Tris pH 7.5, 10% glycerol) and reloaded onto a PC column equilibrated with Buffer D supplemented with 100 mm NaCl. After copious washing with Buffer E supplemented with 100 mm NaCl, the protein was eluted with Buffer F (20 mm phosphate buffer pH 7.8, 500 mm NaCl) and loaded onto a Ni-NTA column equilibrated with the same buffer. The bound protein was washed with Buffer F supplemented with increasing concentrations of imidazole, followed by elution with Buffer F containing 400 mm imidazole. Fractions containing Polλ were diluted five-fold with Buffer D, reloaded onto a PC column, and eluted with Buffer D supplemented with 500 mm NaCl. The resulting protein solution was adjusted by addition of glycerol and bovine serum albumin to 50%, and 0.1 mg mL^−1^, respectively.

pRSET-Polβ^43^ was expressed in BL21(DE3)-pLysS cells in LB medium at 37°C to an OD_600nm_ of 0.5. Protein expression was induced by addition of 1 mm IPTG and continued at 37°C for 3 hours. Cells were pelleted by centrifugation and frozen at −80°C. The cell pellets were thawed and resuspended in Buffer G (25 mm Tris-HCl pH 7.5, 1 mm EDTA, 1 mm PMSF, 10 mm Na_2_S_2_O_5_, and 1 mg L^−1^ pepstatin A) supplemented with 500 mm NaCl, lysed by sonication, and clarified by centrifugation. The resulting supernatant was diluted with Buffer H (Buffer G supplemented with 75 mm NaCl) and loaded onto a Q-sepharose column connected in series with a ssDNA-cellulose column equilibrated with Buffer H. Polβ passes through the Q-sepharose and is retained in the ssDNA-cellulose matrix. After loading, the Q-sepharose column was detached from the ssDNA-cellose, at which point the ssDNA-cellulose is further washed with Buffer H, and bound proteins are eluted by a linear gradient to Buffer I (Buffer G supplemented with 1 m NaCl). Fractions containing Polβ were dialyzed against Buffer H, passed through a 0.22 μm filter and loaded onto a Mono S HR 10/10 column equilibrated with Buffer H. Bound proteins were eluted with a linear gradient to 1 M NaCl (Buffer I).

Both 300 bp DNA substrates were generated by PCR amplification of a fragment of the mouse Jk1 locus in the presence of Cy5-labeled dCTP, followed by restriction digestion of sites appended to this common core. The 3ʹ GCG overhang-containing substrate (complementary DSB) was generated by amplification with primers 5ʹ-TTTTTGCCACGCTGGCTTAGCTGTATAGTCAGGGA-3ʹ and 5ʹ-CACCTGCCTCGCTGGCACACCCATCTCAGACTGGC-3ʹ, and digested with BglI. The 3ʹ-G overhang substrate (noncomplementary DSB) was generated by amplification with primers 5ʹ-CAAGTGGACCACATGTCTTAGCTGTATAGTCAGGGAAATC-3ʹ and 5ʹ-CCGCCGACGCCATGTCACACCCATCTCAGACTGGCTACCC-3ʹ, and digested with AhdI. Complete digestion was validated by electrophoresis, and substrates further purified with a QIAquick cartridge. End-joining reactions were performed with 1 nm substrate, 10 nm Ku, 20 nm XRCC4-Ligase IV complex, and 12.5 nm polymerase in a reaction with 10% polyethylene glycol, 150 mm KCl, 25 mm Tris pH 7.5, 10 μm each dNTP, 100 μm each rNTP, and 100 ng plasmid DNA for 10 minutes. Ligation products were resolved using 5% native PAGE electrophoresis before gel imaging and qualitative analysis.

### Reporting summary

Further information on research design is available in the Nature Research Reporting Summary linked to this article.

## Supplementary information

Reporting Summary

## Data Availability

Atomic coordinates and structure factors have been deposited in the Protein Data Bank (www.pdb.org) with ID codes 6WIC [10.2210/pdb6WIC/pdb], 6WID [10.2210/pdb6WID/pdb], and 6WIE [10.2210/pdb6WIE/pdb]. Source data are provided with this paper. Other data supporting the findings of this study are available from the corresponding author upon reasonable request. Source data are provided with this paper.

## Source data

Source Data
